# Helminth extracellular vesicles: Interactions with the host immune system

**DOI:** 10.1016/j.molimm.2021.06.017

**Published:** 2021-09

**Authors:** Claire Drurey, Rick M. Maizels

**Affiliations:** Wellcome Centre for Integrative Parasitology, Institute of Infection, Immunity and Inflammation, University of Glasgow, UK

**Keywords:** Helminths, Immunomodulation, Vaccines

## Abstract

As long-lived parasites, helminths depend upon immunomodulation of their hosts for survival. The release of excretory-secretory (ES) products, including proteins, lipids and RNAs is how successful host manipulation is achieved. It has recently been discovered that the ES products of helminths contain extracellular vesicles (EVs), with every species investigated found to secrete these lipid-bound structures. EVs are perfect for packaging and delivering immune modulators to target cell types. This review outlines the research carried out on helminth EVs and their constituents thus far, as well as their interaction with components of the mammalian immune system. We discuss how targeting EVs will aid treatment of helminth infection and consider how EVs and their immunomodulatory cargo could be used as therapeutics as we progress through this exciting era.

## Highlights

• Helminth EV release, a new paradigm in host-parasite interactions.
• EV cargo of immune modulators.
• Heterogeneity of EVs in source and targets.
• EVs modulate the epithelium, innate and adaptive immune cells.
• EVs may have therapeutic properties.

## 1 Introducing helminths

Parasitic helminths are macroparasites, many of which inhabit the digestive tract of their hosts. ‘Helminth’ derives from the Greek for ‘worm’, with members sharing this similar form. Despite physical similarities, the group is made up of anciently-diverged phyla; Nematoda (roundworms) and Platyhelminthes (flatworms), the latter subdividing into cestodes (tapeworms) and trematodes (flukes). These parasites often have complex life cycles, requiring both definitive hosts, in which adults reside and sexually reproduce, and intermediate hosts or vectors, in which larvae develop and spread. Many parasites also traffic through multiple sites in their host, for instance the human hookworms, *Necator americanus* and *Ancylostoma duodenale*, enter the human body by penetrating the skin, then move through the bloodstream to the lung before being coughed up, swallowed and reaching their feeding site in the small intestine to mature into egg-producing adults (Hotez et al., 2004). In terms of interaction with the host, it is therefore important to consider the different tissue environments of lifecycle stages.

Parasitic helminths are almost universal in their colonisation of vertebrates, and thus are a health consideration to both humans and species of economically important animals. Around 20 % of the world’s population are infected by soil-transmitted helminths alone (Pullan et al., 2014; Jourdan et al., 2018), with schistosomiasis and the vector-borne helminthiases - lymphatic filariasis and onchocerciasis - adding another 142 million and 87 million people affected globally by helminths (James et al., 2018). Infections are generally long-lived and while direct mortalities are few, morbidities are wide ranging, including anaemia and growth stunting, cognitive defects, and elephantiasis (Pabalan et al., 2018; Pullan et al., 2014). This list increases over time, with *Onchocerca volvulus*, the agent of river blindness, recently being implicated as the cause of nodding syndrome, a form of epilepsy in children (Johnson et al., 2017). Three helminth infections, the trematodes *Opisthorchis viverrini* and *Clonorchis sinensis,*andthe blood fluke*Schistosoma haematobium*, have been classified as Group 1 biological carcinogens, leading to cholangiocarcinoma (bile duct cancer) and squamous cell carcinoma of the bladder (Brindley and Loukas, 2017; Iarc, 2012). Infection with helminths can also affect diseases caused by other agents; recent areas of investigation include the effect of helminth infection on female reproductive health and susceptibility to sexually transmitted diseases (Chetty et al., 2020).

**Fig. 1:**
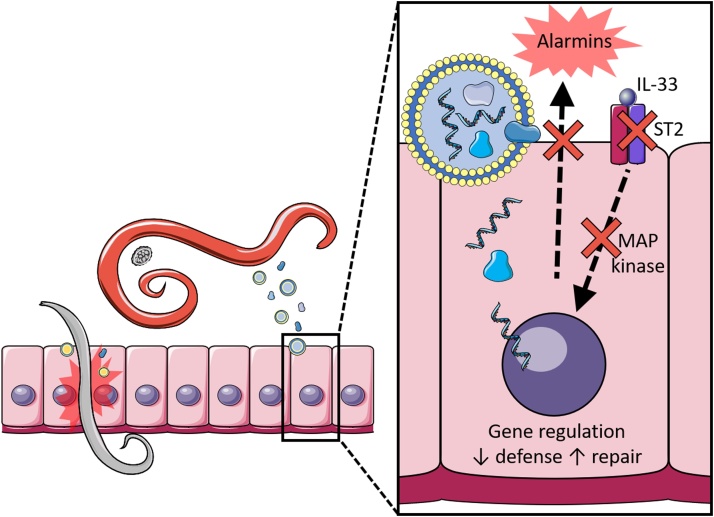
Helminth EVs and the epithelium. Extracellular vesicles are released by all life stages of parasitic helminths, and are taken up by the epithelial cells that form the first layer of defense against them. EVs downregulate the IL-33 receptor subunit ST2 as well as regulators of MAP kinase signalling. EVs induce wound healing pathways and alter immune regulators such as IL-6, resulting in increased epithelial repair and compromised anti-helminth defense. Images are adapted from Servier Medical Art by Servier (http://smart.servier.com/) and modified by the authors under the following terms: Creative Commons Attribution 3.0 Unported (CC BY 3.0).

The duration of helminth infections combined with a lack of replication in their host leads to a reliance on immune evasion for survival, with both nematodes and platyhelminths evolving similar strategies which can act on all phases of the immune response (Maizels et al., 2018). Molecules from the worm released into its environment, termed excretory secretory (ES) products, form the focus of investigation for immunoregulation of the host (Lightowlers and Rickard, 1988; Moreno et al., 2021; Maizels et al., 2018; Hotterbeekx et al., 2021), with most attention so far on the proteins that can be found within this mixture (Harnett, 2014). Further studies into the lipid, glycomic and small molecule components of ES products have revealed that these are also capable of altering the host immune system (Wangchuk et al., 2019; Hokke and Van Diepen, 2017; Van Die and Cummings, 2010). However, a paradigm shift was introduced with the discovery that helminths also release extracellular vesicles (Marcilla et al., 2012; Buck et al., 2014), and indeed such vesicles have been found in ES products of all helminth parasites examined thus far (Tritten and Geary, 2018). These vesicles have potential immunomodulatory functions and could provide a conduit for helminth parasites to deliver fragile cargo like small RNAs through hostile environments, such as the bloodstream or digestive system of the host, into host cells where they can act on intracellular targets (Coakley et al., 2016) (Fig. 1). In fact, many of the proteins identified in ES products may actually be present in or on EVs, with the EV proteomes of the intestinal and liver flukes, *Echinostoma caproni* and *Fasciola hepatica,* accounting for over 50 % of the proteins found in the secretome (Marcilla et al., 2012).

## 2 Helminth extracellular vesicles

Extracellular vesicles (EV) are non-replicative lipid-bilayer delimited particles released from cells and are classified according to size and origin, with subtypes such as exosomes and microvesicles. Exosomes are generally 40−100 nm in size and derive from the endosome via multivesicular bodies (MVB). MVBs fuse with the plasma membrane to release the exosomes to the extracellular environment in a process well described in mammalian systems, involving the endosomal sorting complex required for transport (ESCRT) (Jadli et al., 2020). Though the ESCRT forms the principal machinery driving exosome release, ESCRT-independent pathways have been reported. These include a pathway requiring membrane microdomains formed of the sphingolipid ceramide (Trajkovic et al., 2008), and a pathway involving members of the tetraspanin protein family, including CD63 (Andreu and Yáñez-Mó, 2014). Hybrid mechanisms combining these three systems could also exist (De La Torre-Escudero et al., 2016).

Microvesicles, also known as ectosomes or microparticles, are derived from budding of the plasma membrane itself and range in size from 50−2000 nm (Sedgwick and D’souza-Schorey, 2018). Apoptotic bodies can be much larger, 100−5000 nm, and ‘bleb’ from the surface of cells undergoing apoptosis. Particles that correspond to the size of both exosomes and microvesicles have been identified in the ES of helminths (Cwiklinski et al., 2015; Hansen et al., 2019), though many seem to be exosome-like (Buck et al., 2014; Harischandra et al., 2018). The ESCRT machinery required for MVB formation, as well as proteins that participate in microvesicle formation, are conserved in helminths including species where EV release has not yet been reported (Bennett et al., 2020b), so helminths certainly seem equipped to make and release EVs of either class. Difficulties in differentiating exact cellular origin lead to the use of the umbrella term ‘EV’. Interestingly, 55 EV biogenesis proteins were found in EVs released by platyhelminths, whereas only 7 were found in those released by nematodes (Bennett et al., 2020b), hinting at differences in production between the two groups.

Not only the cellular source of EVs, but their route of secretion from the helminth is still not fully characterised. So far, the main site of discovery has been the gut of the parasite. TEM of the adult intestine of the murine model nematode, *Heligmosomoides polygyrus* identified vesicle budding from the epithelial surface, with structures similar in size to exosomes at the anterior opening (Buck et al., 2014). Concurrent proteomic analysis identified EV components matching proteins from the apical surface of the intestinal epithelium, further supporting this site of secretion. miRNA content in EVs from the barber’s pole worm, *Haemonchus contortus,* suggest the gut as the source in L4 larvae but not in adults (Gu et al., 2017). Investigation of the miRNA in *Ascaris suum*, a nematode parasite of pigs, did not draw a conclusion about whether EVs were released from the anus or secretory pore due to similarities between EVs from ES products, intestinal tissue and *A. suum* body fluid (Hansen et al., 2019). Staining for EV components suggested the excretory-secretory pore of the filarial nematode *Brugia malayi* as the site of vesicle release in microfilariae (Harischandra et al., 2018). Again, platyhelminths show differences as EV release is seen directly from their tegument, a syncytium covering their bodies used for both protection and absorption of nutrients, which in nematodes is replaced by a collagenous extracellular cuticle. In the liver fluke *F. hepatica*, further evidence suggests that in addition to exosomes shed from the tegument (as indicated by their size and the presence of MVBs), most EVs are released through the gut and then oral sucker, with additional output from the protonephridial system through the parasite’s excretory pore (Bennett et al., 2020a).

Though source and means of biosynthesis of helminth EVs are important questions to answer, a bigger question to address is what their physiological function may be. Is their release incidental to a parasite-intrinsic process, or does it fulfil some purpose in host manipulation? EVs act across an extraordinary range of biological systems (Kalluri and Lebleu, 2020), including mammalian immunity, with increasing evidence that EVs enable immune regulation (Zhou et al., 2020). They form a part of the communication between innate and adaptive immune cells; activated T-cells transmit DNA-containing exosomes back to presenting dendritic cells (DCs), further inducing antiviral responses (Torralba et al., 2018). This system can be abused by cancer, leading to immunosuppression (Kugeratski and Kalluri, 2021). Tumour-derived exosomes have been found that induce T-regulatory cells (Tregs), reduce cytotoxicity of natural killer cells and promote tumour-derived monocyte survival, amongst other things (Bebelman et al., 2018). It is no surprise then, that pathogens have also evolved EVs as a means of immunosuppression. The malaria parasite, *Plasmodium berghei,* secretes EVs that are able to inhibit T-cell responses (Demarta‐Gatsi et al., 2019). *Leishmania* exosomes have been found to push monocytes and dendritic cells towards anti-inflammatory phenotypes, increasing Th2 polarisation and so disease (Silverman et al., 2010). There is therefore great interest in how the EVs identified from parasitic helminths may interact with the host immune system, especially as infections are long-lived and mainly asymptomatic with widespread evidence for immune down-modulation.

## 3 EVs – a precious cargo?

The cargo of EVs includes lipids, nucleic acids and proteins; these may be enveloped by the membrane or incorporated within it. Due to their size, there is a high ratio of membrane to cytoplasm, so a high percentage of ‘contents’ may actually be attached to or form a part of the membrane; and unlike a cell all membrane-associated components are peripheral as there are no internal membrane-bound structures. Around 20 % of proteins found within the blood fluke *Schistosoma mansoni* EVs contained transmembrane domains (Kifle et al., 2020b), and 380 surface proteins have been identified from *F. hepatica* EVs (De La Torre-Escudero et al., 2019).

As a result of their biogenesis, there are some components of EVs that are shared across kingdoms. In the case of exosomes, ESCRT and accessory proteins, required for MVB formation, are found regardless of cell type of origin (Colombo et al., 2013). Proteins from the ESCRT pathway have been identified in *F. hepatica* EVs (De La Torre-Escudero et al., 2019). Other exosome markers are proteins from the tetraspanin family, which require the ESCRT-associated protein ALIX to recruit ESCRT-III proteins for their delivery (Larios et al., 2020). Proteins of the tetraspanin family form clusters and interact with other proteins to organise membrane domains, tetraspanin-enriched microdomains (TEMs), and may be the method through which specific proteins can be targeted to EV membranes, as well as aiding adherence of EVs to the membranes of target cells (Andreu and Yáñez-Mó, 2014). CD9 and CD63 are endosome-specific tetraspanins that are used as exosome markers in mammalian systems (Kowal et al., 2016). Antibodies against human CD9 are able to detect tetraspanins in EVs from larvae of the dog tapeworm *Echinococcus granulosus* (Nicolao et al., 2019). CD63 homologues have been identified as components of *F. hepatica* and *Trichinella spiralis* EVs (Cwiklinski et al., 2015; Yang et al., 2020) and antibodies against the parasite protein increased *F. hepatica* EV uptake by macrophages (De La Torre-Escudero et al., 2019).

Microvesicles also contain tetraspanins, due to their biogenesis via outward budding of the cell membrane, which contains clusters of these proteins. Tetraspanins have been found in EVs from the model helminth *H. polygyrus* (Buck et al., 2014) and the blood flukes *Schistosoma* spp (Mekonnen et al., 2020; Samoil et al., 2018) amongst others. As tetraspanins appear ubiquitous amongst EVs, they have been tested as potential vaccine antigens, with tetraspanins from the platyhelminths *E. multilocularis, Opisthorchis viverrini* and *S. mansoni,* inducing protective responses of 30–60 % reductions in parasite burdens in mouse models (Tran et al., 2006; Dang et al., 2012; Chaiyadet et al., 2019). Among the nematodes, mice immunized with tetraspanins from *B. malayi* generated responses able to kill infective larval parasites, although as mice are refractory to infection, overall levels of protection could not be evaluated (Dakshinamoorthy et al., 2013).

As microvesicles bud directly from the plasma membrane, they contain many cell-specific membrane proteins which act as clues as to the cell type of origin. In *H. polygyrus* EVs, many proteins were identified that are associated with the apical membrane of the intestinal tract in the free-living nematode *Caenorhabditis elegans*, supporting their site of release as the gut (Buck et al., 2014). Similarly, some cytoplasmic proteins may indicate provenance, as in the case of a population of extracellular vesicles from *F. hepatica* which contain the protease cathepsin L1, derived from gastrodermal cells of the fluke gut; transmission electron microscopy (TEM) confirmed this source (Cwiklinski et al., 2015).

If a function of parasitic helminth EVs is host immune regulation, then it would be expected that they contain contents outside of the ‘ordinary’ EV cargo. Most analyses of helminth EV content have been at the protein level. Sotillo et al. performed an analysis of released protein datasets and found that while there is no apparent universal EV marker for all helminth species, proteins from the EF-hand family were identified across EVs from cestodes and trematodes, whereas M13 metallopeptidases were present in all nematode EVs (Sotillo et al., 2020). In addition, some proteins are present in both EVs and in the soluble compartment of ES products, which may explain why both can exert parallel immunomodulatory effects; for example, proteins from the SCP/TAPS family are common in EVs from several nematode species, including the murine models *H. polygyrus, Nippostrongylus brasiliensis* and *Trichuris muris* (Buck et al., 2014; Eichenberger et al., 2018b, a), as well as the sheep parasite *Teladorsagia circumcincta* (Tzelos et al., 2016). This family of proteins, also known as venom allergen-like, are known to be secreted by both plant and animal parasitic nematodes to modulate host responses (Wilbers et al., 2018). Interestingly, however, the majority of proteins found in helminth EVs, compared using Pfam accession, were found in only one parasite species (Sotillo et al., 2020). This is perhaps to be expected if EVs are a tool in host interaction, as each helminth has a specific host, and niche within that host, in which to survive. An unanswered question, however, is whether EV proteins fulfil a unique function, or are more effective when delivered as part of the vesicle cargo, than when the same proteins are released into the soluble milieu.

Helminth EVs also contain species of small RNAs, which can play a role in host-helminth interaction (Sotillo et al., 2020; Wu et al., 2019; Fromm et al., 2017). Proteins implicated in RNA-loading into EVs such as argonaute 2 (Ago2) are conserved in helminths, though there are some differences between phyla (Bennett et al., 2020b). Major vault protein (MVP) is conserved solely in platyhelminths and present in their EVs, and there has been a lineage-specific expansion of argonaut proteins (WAGOs) in nematodes (Bennett et al., 2020b). A WAGO from *H. polygyrus* is found associated with small interfering (si)RNAs specifically found within EVs, suggesting a means of selective export (Chow et al., 2019). It is the micro (mi)RNAs that provide some of the biggest clues for immunomodulation, with many found that have homology to their host, some against genes involved in immunity (Zamanian et al., 2015; Buck et al., 2014; Hansen et al., 2019; Zhu et al., 2016; Tritten et al., 2016; Yang et al., 2020; Fromm et al., 2015).

Lipids are also a major group of EV contents, though not as well characterised as the protein and small RNA components. They are a vital part of the vesicle, forming a membrane to protect and keep together the internal cargo. Lipidomic analysis has been undertaken of *H. polygyrus* EVs, which identified enrichment of the ether phospholipids plasmalogens. These are believed to increase rigidity of the membrane, enhancing durability in the intestine (Simbari et al., 2016). It is also suggested that plasmalogens may protect against reactive oxygen species (ROS) or aid fusion with host membranes (Whitehead et al., 2020). Lipids can also be bioactive themselves, contributing to cell uptake and modulating immunity directly (Record et al., 2018; Whitehead et al., 2020). Lipids produced by *S. mansoni* have been found to trigger M2 polarisation of macrophages (Assunção et al., 2017) and activate human eosinophils (Magalhães et al., 2019), with some suggesting that the most effective means of delivery for these lipids is via EV (Coakley et al., 2019). Further investigation of this group of EV cargo is needed to understand the multiple roles lipids may play.

Glycans are another neglected group within helminth EV biology. Both EV proteins and lipids can be post-translationally glycosylated, which is known to be essential for regulation of protein function (Varki, 2017). Glycans have been identified as key players in the regulation of EV uptake, affecting cell tropism of EVs (Williams et al., 2019). Glycans from *F. hepatica* EVs have been profiled using lectin microarrays, showing that their composition is different to that found on the tegument of the fluke (De La Torre-Escudero et al., 2019; Murphy et al., 2020). Removal of EV external glycans using glycosidases blocked their uptake by a macrophage cell line, suggesting a need for these carbohydrates in cellular uptake (De La Torre-Escudero et al., 2019). Glycans may even play a role in biodistribution of helminth EVs; glycosidase treatment of mouse liver EVs led to accumulation in the lung, rather than the liver (Royo et al., 2019). EV glycans from *S. mansoni* are similar to those found in total schistosomula extracts and include ligands for DC-SIGN (CD209), the receptor on dendritic cells needed for EV uptake (Kuipers et al., 2020). This suggests that EV glycosylation is needed for interaction with host immune cells. Other suggested functions of glycans present on the surface of EVs include acting as a shield or source of decoy antigens, and activation or evasion of host complement pathways (Whitehead et al., 2020). However, more research is needed to elucidate the true functions of this group of EV components. Novel proteomic approaches will aid in the identification of glycosylation, as well as other post-translational modifications such as phosphorylation and ubiquitination (Montaño et al., 2021).

## 4 Not all EVs are equal

Recently, investigation of different populations of vesicles from individual helminth species has been undertaken. EVs in ES products are not uniform and have been found to differ markedly in terms of size and content. EVs can be stratified according to size by centrifuging at different speeds, these speeds giving rise to the names of the sub-populations. Although a central group of proteins are shared within EVs of different sizes, such as some tetraspanins and other proteins involved in vesicle biogenesis, binding and trafficking, different protein cargoes have been found (Kifle et al., 2020b; Cai et al., 2021; De La Torre-Escudero et al., 2019). Smaller EVs from *E. granulosus* were found to be internalised by hepatic cells faster than larger vesicles, and also contained more proteins that were recognised by infected human serum, suggesting that different populations of EVs have distinct roles in host interactions (Cai et al., 2021). Analysis of *F. hepatica* EV surface proteins identified differences between 15k and 120k populations of EV, with cathepsin L1 specific for 15k and CD63 enriched in 120k EVs (De La Torre-Escudero et al., 2019; Cwiklinski et al., 2015). This correlates to differences in cellular origins of the EV sub-populations; with the larger 15k EVs released from gastrodermal cells lining the gut and 120k EVs seen beneath the gastrodermis, seemingly within the liver fluke excretory system (De La Torre-Escudero et al., 2019). Physically ligating both the oral sucker and excretory pore of *F. hepatica* blocked the release of the 120k EVs but not the 15k population (Bennett et al., 2020a). A large amount of morphological variability is also seen in EVs from *F. hepatica* produced throughout the parasites lifecycle, from eggs, juveniles and adults (Sánchez-López et al., 2020). This included vesicles with electron-dense ‘spikes’ protruding from their membrane, possibly formed of proteins that facilitate membrane fusion, as they do in viruses (Harrison, 2015).

Vesicle contents have also been found to differ depending on gender and lifecycle stage of the helminth. The EV proteome of *B. malayi* is both stage and sex specific – more than three times as many proteins were identified in female than male EVs (Harischandra et al., 2018). EVs are released throughout the *B. malayi* lifecyle, from microfilariae to adults that reside in the lymphatics (Harischandra et al., 2018), however EVs are most abundant from L3 larvae, the stage which has to navigate from the mosquito, through human skin to the lymphatic system (Zamanian et al., 2015). *H. contortus* adult and L4 EVs are enriched for different groups of miRNAs, perhaps reflecting their slightly different niches within the abomasum (Gu et al., 2017). The same is true in *Dirofilaria immitis*, where Tritten et al. found that miRNAs are enriched differentially based on both lifecycle stage and sex, though this was from whole ES products rather than EVs alone (Tritten et al., 2016). Interestingly, the pig nematode *A. suum* L3 and L4 larval EVs contain more unique miRNAs than in the adult (Hansen et al., 2019). This increase of cargo in larval stages may be due to the need to manipulate a changing host environment during migration, compared to adults which take up fixed residence in a specific tissue.

Nearly all EVs from helminths are collected from parasites cultured in vitro and purified from conditioned media; in a rare exception, Zhou et al. were able to harvest EVs from the hydatid cyst fluid in *E. granulosus* patients, and compare them with those generated by the protoscolex stage of the parasite in vitro (Zhou et al., 2019). Although morphologically similar, the two sets of EVs were divergent in protein content and abundance, with the *ex vivo* cyst EVs containing over 1000 protein species, most of which were absent from the protoscolex vesicles; however, the proteins detected in the latter were mostly present in the cyst vesicles. Hence, in this study the *in vitro* collected EVs may not represent the full spectrum of components expressed *in vivo*.

## 5 Helminth EVs and immunity

### 5.1 EVs and the epithelium

The first point of contact with the host for most helminths is an epithelium of some kind. This is the tissue that alerts the immune system to the presence of the worm, through the release of alarmins such as TSLP, IL-25 and IL-33 (Oyesola et al., 2020; Peterson and Artis, 2014). Intestinal epithelial cell uptake of EVs has been demonstrated with vesicles from the nematodes *H. polygyrus, N. brasiliensis* and *T. muris* (Buck et al., 2014; Eichenberger et al., 2018b, a) and the trematode *E. caproni* (Marcilla et al., 2012). *H. polygyrus* EV treatment of mouse epithelial cell lines led to downregulation of an IL-33 receptor subunit (known as ST2) and *Dusp1,* a regulator of MAPK signalling and key component of the anti-inflammatory response (Buck et al., 2014). The presence of miRNAs complementary to the 3’ untranslated region of *Dusp1* in the EVs, and their ability to suppress *Dusp1* expression suggests a mechanism for this gene regulation (Buck et al., 2014). Interestingly, two distinct *H. polygyrus* ES products are known to interfere with IL-33 signalling, firstly *H. polygyrus* Alarmin Release Inhibitor (HpARI) which binds IL-33, preventing its release from dying cells (Osbourn et al., 2017), and a second protein that binds to and inhibits the IL-33 receptor ST2, hence its name Binds Alarmin Receptor and Inhibits (HpBARI) (Vacca et al., 2020). It is likely that both proteins may be present in EVs which, added to downregulation of the receptor itself, identifies IL-33 signalling as a key target of helminth immunoregulation.

A special case of helminth-epithelial interaction is in *O. viverrini*, a trematode infecting the bile duct of humans. Parasite EVs are taken up by human cholangiocytes, the cells of the bile duct epithelium, promoting their proliferation and inducing wound healing pathways (Chaiyadet et al., 2015). The suckers of the worm induce mechanical damage of the epithelium, so perhaps by promoting repair mechanisms, the parasite has adapted to maintain the health of the host. However, relentless cell proliferation induced by *O. viverrini* ES products alongside chronic immunopathology due to the presence of the worm can frequently lead to malignant cholangiocarcinoma (Zheng et al., 2017). A parallel may exist with adult schistosomes, which live in the vasculature, and so are in contact with the vascular endothelium. Similar to *O. viverrini*, chemokines involved in tissue repair are also upregulated by *S. mansoni* EVs when they are taken up by a human endothelial cell line, suggesting a means by which the worm encourages homeostasis in the host (Kifle et al., 2020a). Immune regulatory genes, such as IL-6, are also differentially regulated, as well as genes involved in blood clotting and vasodilation, perhaps aiding this blood-feeding helminth (Kifle et al., 2020a).

Helminths that migrate through tissues, such as Schistosomes which need to penetrate the skin, are known to release cathepsins and other proteases to aid in infiltration (Grote et al., 2018). Cathepsins are even found within and on the surface of EVs (De La Torre-Escudero et al., 2019; Zamanian et al., 2015; Marcilla et al., 2012; Tzelos et al., 2016). Arguably, EVs may also contain products that when delivered to cells within the epithelium counteract and repair the damage caused by the larvae as they pass through. For example, although migration of hookworm larvae through the lung causes a large amount of both mechanical and enzymatic damage, there is a remarkable amount of rapid repair of the pulmonary pathology (Schwartz et al., 2018). EVs may be the means through which helminths regulate this. *N. brasiliensis* EVs suppressed inflammatory cytokines IL-6 and IFNγ, as well as upregulating the anti-inflammatory cytokine IL-10 in mouse models of colitis (Eichenberger et al., 2018a). Reduction of immune signalling will also aid in returning the system to homeostasis quickly, keeping the host healthy, which is needed if long-lived infections are to be successful.

In filarial nematodes, microfilariae circulate through the peripheral blood of the host, encountering a wide range of host cells. EVs released into the blood can be substantial – a single *B. malayi* microfilaria can release around 1.9 × 10^4^ EVs in an hour (Harischandra et al., 2018), leading to high levels of EV in the serum. miRNAs from the murine filarial model *Litomosoides sigmodontis* have been found in host serum and macrophages, the most abundant of which match those found in EVs (Buck et al., 2014; Quintana et al., 2019). Some non-filarial helminths also spend some time in the circulation; for example, parasite EVs can be isolated from *Schistosoma* spp. infected human sera, and miRNAs from these have been suggested as a tool for diagnosis of schistosomiasis (Meningher et al., 2017). Encapsulation of small RNAs in parasite EVs would prevent their degradation in the blood, enabling them to be delivered across the body and have organism-wide effects. This may be particularly important for blood-migrating larval stages to prevent systemic inflammation in response to the invaders.

### 5.2 EVs and phagocytes

The immune cells at the centre of helminth EV studies have been phagocytes, specifically macrophages. Macrophage cell lines have been shown to take up EVs from *B. malayi, H. polygyrus* and *F. hepatica* (Coakley et al., 2017; Zamanian et al., 2015; De La Torre-Escudero et al., 2019). EVs from the zoonotic marine nematode *Anisakis* spp are taken up by a human monocyte cell line (Boysen et al., 2020). Though perhaps it is to be expected that phagocytic cells will constitutively internalise EVs from the environment around them, it has been shown that uptake differs from classical endocytosis induced by antibodies. Antibodies against *S. mansoni* tetraspanins actually block uptake of both exosome- and microvesicle-like EVs into a human monocyte cell line (Kifle et al., 2020a). Coakley et al. found murine macrophages took up EVs from *H. polygyrus* via phagocytosis or endocytosis into distinct foci in the cytoplasm suggesting an endosomal location, leading to inhibition of both type 1 (M1) and type 2 (M2) macrophage activation (Coakley et al., 2017). Significantly, when antibodies against the EVs were added, uptake was enhanced but EVs were redirected into lysosomes, and suppression of macrophage function was no longer seen (Coakley et al., 2017). Hence the mode of uptake critically determines the intracellular fate of the vesicles. In macrophages, *H. polygyrus* EVs suppressed expression of the IL-33 receptor subunit ST2, echoing earlier findings that the same EVs were able to suppress ST2 expression in innate lymphoid cells (ILCs) in vivo (Buck et al., 2014). Blocking the IL-33 receptor effectively leaves cells blind to the damage caused by migrating and feeding helminths, preventing activation of immune cells.

Alteration of phagocyte activation is a widespread feature of helminth EVs. RAW264.7 macrophages become skewed towards an M1-like phenotype when cultured with EVs from adult *S. japonicum* (Wang et al., 2015). EVs from *B. malayi* infective L3 larvae also induce an M1 phenotype when they are taken up by a murine macrophage cell line (Zamanian et al., 2015). This is in contrast to live *Brugia* and ES products, which stimulate an M2 phenotype in macrophages (Sharma et al., 2018; Semnani et al., 2011). EVs are therefore a discrete portion of helminth secreted products, with demonstrably separate actions to other constituents of the milieu. Moreover, the encasement within a lipid membrane may allow delivery of cargo to specific cells such as leukocytes, in contrast to products that are directly secreted.

**Fig. 2:**
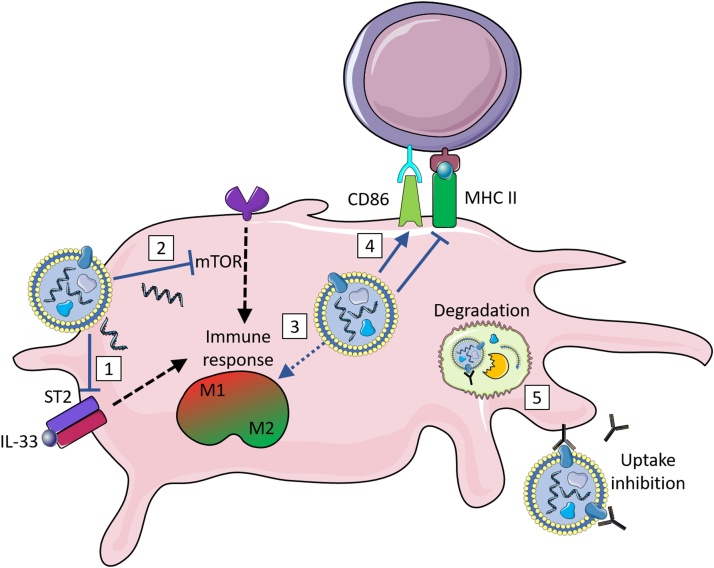
Helminth EVs and myeloid cells. Helminth extracellular vesicles are taken up by myeloid cells, interfering with innate defense pathways. (1) EVs from *H. polygyrus* are able to downregulate the IL-33 receptor subunit ST2, reducing their ability to respond to this cytokine. (2) EVs from *B. malayi* contain miRNAs that target components of the mTOR signalling pathway, downregulating it. These effects, as well as others yet characterised, combine to alter the activation state of macrophages. (3) EVs from *S. japonicum* and *B. malayi* induce M1 polarisation, whereas *H. polygyrus* EVs inhibit both M1 and M2 polarisation. (4) Helminth EVs interfere with the expression of components involved in antigen presentation and lymphocyte activation. *E. granulosus* EVs cause a decrease in MHC II in dendritic cells. *E. granulosus* and *F. hepatica* EVs both increase the costimulatory marker CD86 in dendritic cells. (5) Antibodies raised against helminth EVs prevent their action, either by preventing uptake altogether, or leading to their uptake into a degradative pathway in the lysosome. Images are adapted from Servier Medical Art by Servier (http://smart.servier.com/) and modified by the authors under the following terms: Creative Commons Attribution 3.0 Unported (CC BY 3.0).

EVs from the microfilariae of *B. malayi* are internalised by both human monocytes and dendritic cells (DCs), where they downregulate the signalling pathway of mammalian Target Of Rapamycin (mTOR)(Narasimhan et al., 2016; Ricciardi et al., 2021). This is achieved via the presence of miRNAs that target the mTOR pathway (Ricciardi et al., 2021). The mTOR pathway is a central regulator in the effector response of innate immune cells, and targeting this pathway affects polarisation, migration, cytokine response and antigen presentation in one (Weichhart et al., 2015). *E. granulosus* EVs are also taken up by DCs, inducing their maturation and an unconventional activation profile, with an increase in CD86, required for costimulation of T-cells, but a decrease in MHCII, needed for antigen presentation (Nicolao et al., 2019). Similar results were shown using *F. hepatica* EVs to stimulate DCs, with increased costimulatory markers, including CD86, but lacking the activation marker ICAM-1 needed for long lasting T-cell interaction (Murphy et al., 2020). DCs exposed to *F. hepatica* EVs went on to inhibit antigen-specific production of the T-cell growth factor IL-2 by T-cells, and did not activate the Th2 responses normally seen in response to *F. hepatica* infection (Murphy et al., 2020). Targeting antigen presenting cells such as DCs in this way aids the parasite in blocking antigenic presentation and prevents activation of the next arm of the immune system, adaptive immunity (Fig. 2).

### 5.3 Helminth EVs and adaptive lymphocytes

Though helminth EVs can influence adaptive immunity via regulation of antigen presenting cells, there is also evidence that EVs are able to manipulate lymphocytes directly. Murine peripheral blood mononuclear cells (PBMCs) cultured with EVs from *E. granulosus* showed inhibition of CD4^+^ and CD8^+^ T cell proliferation, as well as release of inflammatory cytokines, and of IL-10 (Zhou et al., 2019). On the other hand, culture of *T. spiralis* EVs with human PBMCs induced IL-6 and IL-10 release (Kosanović et al., 2019). Although tested on different host cells, it is clear that EVs from different helminth species can have different actions and opposing targets. In the case of *T. spiralis,* the effects of EVs would promote parasite survival: IL-10 is an anti-inflammatory regulatory cytokine, able to enhance the Th2 response but also increase T-regulatory cell (Treg) expansion so differing regulation of this cytokine may be needed dependent on life stage and location in the host. IL-6 is known to limit the Th2 response in helminth infection (Smith and Maizels, 2014), perhaps via expansion of Tregs with enhanced suppressive capacity (Hagenstein et al., 2019).

Manipulation of the host immune response via Tregs could be a general target for EVs. Treg expansion would amplify anti-inflammatory signals more broadly than targeting effector T cells directly, and would allow protection of the host from potentially pathogenic immune responses as well as enabling the parasite to survive for longer (White et al., 2020). Accordingly, EVs from *T. spiralis* were able to expand Tregs in a colitis model in vivo (Yang et al., 2020). Moreover, *H. polygyrus* EVs contain a Transforming growth factor (TGF)-β mimic (TGM) protein (Buck et al., 2014), which induces naive peripheral T cells to convert to suppressive Foxp3^+^ Tregs (Johnston et al., 2017). Other EV cargoes include proteins known to modulate T cells, for instance EVs of *E. granulosus* larvae contain basigin, also known as CD147, which regulates lymphocyte responsiveness, inhibiting T-cell proliferation (Nicolao et al., 2019).

The central feature of adaptive immunity is specific recognition of pathogen antigens through B cell and T cell receptors against defined epitopes. However, identification of potential epitopes that the adaptive immune system can target is still at a very early stage. On the one hand, membrane-bound proteins exposed on the EV surface area could present many opportunities for recognition. Antibodies against the surface lead to prevention of uptake, or uptake into degradative pathways, rendering their immunosuppression null (Coakley et al., 2017; Chaiyadet et al., 2019). Studies have found vaccination with EVs has provided some protective immunity against challenge with helminth parasites (Chaiyadet et al., 2019; Coakley et al., 2017; Trelis et al., 2016; Shears et al., 2018). Antibodies against the tetraspanins found on EV surfaces are also able to provide protection against *S. mansoni* in mice (Mekonnen et al., 2020). In fact, tetraspanins from *E. multilocularis, B. malayi* and *O. viverrini* have been considered as vaccine targets (Dakshinamoorthy et al., 2013; Dang et al., 2012; Phung et al., 2019), though in the case of platyhelminth parasites tetraspanins are also found on the tegumental surfaces, so the target of immunity may not be the EVs.

On the other hand, the levels of immunity induced by EVs vary. No decrease in worm burden was seen after vaccination without adjuvant for the trematode *E. caproni,* despite reduction in eggs (Trelis et al., 2016), compared to 80 % reduction in worm burden in the case of *H. polygyrus* vaccination with alum as an adjuvant (Coakley et al., 2017). The nature of EVs means that proteins that may be immunogenic are protected inside the lipid membrane, preventing uptake by antigen presenting cells. In infections, the host may therefore not naturally generate antibodies against EV components; the need for adjuvants in successful vaccinations supports this. However, antigens used for immunodiagnosis of echinococcosis and cysticercosis were found among proteins identified in EVs from the cestodes *E. multilocularis* and *Taenia crassiceps*, suggesting that at least some proteins are processed and presented to the adaptive immune system in these infections (Ancarola et al., 2017). IgA and IgG in sera from *T. circumcincta-*infected sheep bound to components of EV-enriched ES products also suggesting the presence of some antigens (Tzelos et al., 2016).

There is also a history of EV proteins having been selected as vaccine antigens by other criteria. Alongside the tetraspanins, ectoenzymes such as H11 and metalloproteases (MEPs) are also found in EVs (Tzelos et al., 2016; Buck et al., 2014). These were developed as vaccine candidates against *H. contortus* many years before EVs were discovered in this parasite (Andrews et al., 1995), on the premise that they were “hidden antigens” located on the worm intestinal epithelium, that would be neutralised by antibodies ingested with the parasite’s blood meal (Nisbet et al., 2016). While this may well be the mode of action, it is now possible that vaccine-elicited antibodies block the function of EVs which, derived from the worm intestine, express the same antigens on their membranes. Currently, the *H. contortus* vaccine is known commercially as “Barbervax”, and is in use on livestock (Smith, 2014). Although this vaccine does confer protection against *H. contortus* infection, it is short-lived due to a failure to elicit long-term memory responses, indicating either that worm gut proteases are not accessed and presented to induce a memory response, or that EVs are intrinsically poorly immunogenic or evade antigen presentation in other ways.

Tetraspanins and proteases are found in EVs across many different species of helminth, suggesting that vaccines utilising them as antigens could potentially be pan-specific. This would be useful for human helminth infections, as areas endemic for diseases like human hookworm and schistosomiasis overlap, with coinfection common (Hotez et al., 2008). Targeting a functionally constrained and conserved antigen required for vesicle biogenesis would also prevent the evolution of resistance, as well as preventing EVs manifesting the immunomodulation described above (Drurey et al., 2020).

### 5.4 Targeting EVs for chemotherapy of helminth infection

In addition to using components of helminth EVs as vaccine targets, they may also be targeted directly to prevent their immunomodulatory functions. Drugs can be used to target their specific means of biogenesis. Bennett et al. used the chemical inhibitor of neutral sphingomyelinases GW4869 to successfully repress the release of 120 K EVs from *F. hepatica in vitro* (Bennett et al., 2020a). Sphingomyelinases are needed to convert sphingomyelin to ceramide, which drives ESCRT-independent EV biogenesis via membrane curvature (Verderio et al., 2018). Use of GW4869 also altered the tissues of the liver fluke, with a highly vacuolated tegumental syncytium and parenchymal tissue, suggested to be due to the accumulation of sphingomyelin in the cells (Bennett et al., 2020a). Treatment with the inhibitor was non-lethal in vitro, but it remains to be seen whether this inhibition of EV release would be able to reduce parasite survival *in vivo*.

Ivermectin, a drug already used widely to treat helminth infections in both humans and animals, has been found to inhibit EV release in *B. malayi, D. immitis* and *A. suum* (Harischandra et al., 2018; Loghry et al., 2020). Other anti-helminth drugs, including albendazole and levamisole, do not show inhibition of EV secretion, and no effect of ivermectin was seen on a *D. immitis* strain that has resistance to the drug, suggesting a specific mode of action for ivermectin (Loghry et al., 2020; Harischandra et al., 2018). Ivermectin is suggested to work via activation of glutamate-gated chloride channels, leading to hyperpolarisation of cell membranes and so paralysis, however ivermectin can also affect other pathways and its mechanism of action is not fully understood (Laing et al., 2017). Effects on EV secretion by ivermectin are seen at far lower concentrations than that needed for motility effects, and closer to the concentrations found after treatment *in vivo* (Loghry et al., 2020). This suggests that a therapeutic mode of action of ivermectin could involve inhibition of helminth EV secretion, preventing immunomodulation of the host.

### 5.5 Helminth EVs in vivo – anti-inflammatory therapeutic potential?

A major theme in helminth research is the possibility that helminth products or their derivatives could successfully treat inflammatory disorders such as asthma or colitis (Ryan et al., 2020; Maizels et al., 2018). Hence, the observed immunomodulation by helminth EVs with a negative impact in the context of infection, may be transformed into useful therapeutic agents. Indeed, helminth EVs applied to models of inflammation have already been shown to alleviate symptoms, with the most common models used being DSS- or TNBS-induced colitis, as a model of inflammatory bowel disease (IBD). *F. hepatica* EVs are able to prevent DSS-induced colitis, reducing both clinical symptoms and histological alterations (Roig et al., 2018). A decreased level of the pro-inflammatory cytokines IL-6 and TNF was measured, but no changes in IL-10 levels were seen, which along with protection being maintained in Rag1^−/−^ mice, lacking B and T cells, suggests no role for Tregs (Roig et al., 2018). The most likely agents of protection were suggested to be macrophages, due to the characterised effects of helminth infections activating them to an anti-inflammatory M2 type.

Investigations with *T. spiralis* EVs have, however, revealed roles for both macrophages and Tregs. Treatment of mice with *T. spiralis* EVs reduces the severity of both DSS- and TNBS-induced colitis (Yang et al., 2020; Gao et al., 2021). Lower levels of the pro-inflammatory cytokines IL-1β and TNF were measured in colonic tissue, alongside an increase in the anti-inflammatory Th2 cytokines IL-4 and IL-13. In contrast with the *F. hepatica* study, levels of IL-10 were also increased, agreeing with results found from EV treatment of cells *in vitro* (Kosanović et al., 2019). Increased expression of Foxp3 was found in colonic tissue, with an increase in Foxp3+ Tregs in the MLN, suggesting a role for Tregs in reduction of TNBS-induced colitis (Yang et al., 2020). In the DSS model, EVs were found to inhibit M1 macrophage polarisation, with an increased infiltration of M2 macrophages into the colon believed to be responsible for tissue repair, lower clinical activity and histopathological scores (Gao et al., 2021). In support of this, macrophages alone, transferred after activation to M2 by *T. spiralis,* are enough to inhibit DSS-induced colitis (Kang et al., 2019).

Both these *T. spiralis* studies used injection with PBS alone as a control to EV treatment in intraperitoneal injections, so it cannot be ruled out that EVs could be acting via antigenic diversion rather than actively reducing inflammation via immunomodulation. However, EVs from the model hookworm *N. brasiliensis* but not the whipworm *T. muris* were able to protect against TNBS- induced colitis in mice, suggesting specificity (Eichenberger et al., 2018a). Treatment with *N. brasiliensis* EVs led to a reduction in levels of pro-inflammatory cytokines, including IL-1β and IL-6, alongside increased IL-10 levels, which were higher than that seen for whole ES product treatment (Eichenberger et al., 2018a). Differences in miRNA content between EVs from the two species were suggested to be the reason behind the effectiveness of *N. brasiliensis* EVs, with more cytokine-encoding genes predicted to be targeted by this species (Eichenberger et al., 2018a).

There is also evidence that helminth EVs reduce inflammation in non-intestinal tissues. *H. polygyrus* EVs reduce inflammation in the *Alternaria* lung allergy model, which induces IL-33 release leading to lung eosinophilia. EV treatment led to a reduction in eosinophils and suppressed IL-5 and IL-13 expression in ILCs (Buck et al., 2014). Expression of the IL-33 receptor ST-2 was reduced in these cells, which is also found in macrophages exposed to these EVs (Coakley et al., 2017). *H. polygyrus* is a model for soil-transmitted helminths and does not migrate through the murine lung. The ability of EVs from this species to reduce inflammation in a tissue distal to its host niche suggests that helminth EVs have the potential for use as anti-inflammatory therapeutic agents throughout the body.

Medicine could also learn from the packaging of the EV itself, as a way of content delivery to specific cell types. Overexpression of the EV marker CD63 fused to OVA encouraged its packaging into EVs which were able to induce higher immune responses against OVA (Kanuma et al., 2017). Artificial spherical vesicles known as liposomes have already shown promise as oral vaccines in the *N. brasiliensis* mouse model (Bartlett et al., 2020), and adding components found in EVs could make them more immunogenic. It is an interesting thought that the very weapons helminths use to control their hosts could be turned against them and used for their downfall. Conversely, rather than using EVs to induce stronger immunity, by including immunomodulatory proteins from helminths as the cargo, they could be used as targeted anti-inflammatories (Mardahl et al., 2019). Further research is needed on both components involved in vesicle targeting and exact means of immunomodulation, but this is an exciting possibility.

## 6 Concluding remarks

Dependent on their hosts for survival, helminths are uniquely placed for the evolution of products with immunomodulatory properties. Extracellular vesicles contained within helminth excretory/secretory products are one of the strategies used by the parasite to manipulate multiple layers of the immune system. We are only just beginning to understand the functions of the varied proteins, lipids, carbohydrates and RNAs packaged within EVs and their effects on the host. Future research is needed to characterise the specific actions of EV cargo on cells of the immune system. Not only are these findings fascinating from a host-parasite interaction perspective, but they may also inform therapeutic strategies for both helminth infection and inflammatory disease.

## Author statement

**Claire Drurey:** Conceptualization; Writing - Original Draft; Writing - Review & Editing.

**Rick Maizels:** Conceptualization; Writing - Review & Editing; Funding acquisition.
